# Association of Male Hypogonadism With Risk of Hospitalization for COVID-19

**DOI:** 10.1001/jamanetworkopen.2022.29747

**Published:** 2022-09-02

**Authors:** Sandeep Dhindsa, Cosette Champion, Ekamjit Deol, Matthew Lui, Robert Campbell, Jennifer Newman, Aparna Yeggalam, Srikanth Nadella, Vaishaliben Ahir, Ekta Shrestha, Thomas Kannampallil, Abhinav Diwan

**Affiliations:** 1 Division of Endocrinology, Diabetes and Metabolism, St Louis University School of Medicine, St Louis, Missouri; 2 Department of Medicine, Washington University School of Medicine in St Louis, St Louis, Missouri; 3 School of Medicine, St Louis University, St Louis, Missouri; 4 Department of Anesthesiology, Washington University School of Medicine in St Louis, St Louis, Missouri; 5 Center for Cardiovascular Research, Washington University School of Medicine in St Louis, St Louis, Missouri; 6 Department of Medicine, Washington University School of Medicine in St Louis, St Louis, Missouri; 7 Department of Cell Biology and Physiology, Washington University School of Medicine in St Louis, St Louis, Missouri; 8 Department of Obstetrics and Gynecology, Washington University School of Medicine in St Louis, St Louis, Missouri; 9 Division of Cardiology, Medicine Service, John Cochran Veterans Affairs Medical Center, St Louis, Missouri

## Abstract

**Importance:**

Male sex is associated with severe COVID-19. It is not known whether the risk of hospitalization differs between men with hypogonadism, men with eugonadism, and those receiving testosterone therapy (TTh).

**Objective:**

To compare COVID-19 hospitalization rates for men with hypogonadism who were not receiving TTh, men with eugonadism, and men receiving TTh.

**Design, Setting, and Participants:**

This cohort study was conducted in 2 large academic health systems in St Louis, Missouri, among 723 men with a history of COVID-19 who had testosterone concentrations measured between January 1, 2017, and December 31, 2021.

**Exposures:**

The primary exposure was gonadal status (hypogonadism, eugonadism, and TTh). Hypogonadism was defined as a total testosterone concentration below the limit of normal provided by the laboratory (which varied from 175 to 300 ng/dL [to convert to nanomoles per liter, multiply by 0.0347]).

**Main Outcomes and Measures:**

The primary outcome was rate of hospitalization for COVID-19. Statistical adjustments were made for group differences in age, body mass index, race and ethnicity, immunosuppression, and comorbid conditions.

**Results:**

Of the 723 study participants (mean [SD] age, 55 [14] years; mean [SD] body mass index, 33.5 [7.3]), 116 men had hypogonadism, 427 had eugonadism, and 180 were receiving TTh. Men with hypogonadism were more likely than men with eugonadism to be hospitalized with COVID-19 (52 of 116 [45%] vs 53 of 427 [12%]; *P* < .001). After multivariable adjustment, men with hypogonadism had higher odds than men with eugonadism of being hospitalized (odds ratio, 2.4; 95% CI, 1.4-4.4; *P* < .003). Men receiving TTh had a similar risk of hospitalization as men with eugonadism (odds ratio, 1.3; 95% CI, 0.7-2.3; *P* = .35). Men receiving inadequate TTh (defined as subnormal testosterone concentrations while receiving TTh) had higher odds of hospitalization compared with men who had normal testosterone concentrations while receiving TTh (multivariable adjusted odds ratio, 3.5; 95% CI, 1.5-8.6; *P* = .003).

**Conclusions and Relevance:**

This study suggests that men with hypogonadism were more likely to be hospitalized after COVID-19 infection compared with those with eugonadism, independent of other known risk factors. This increased risk was not observed among men receiving adequate TTh. Screening and appropriate therapy for hypogonadism need to be evaluated as a strategy to prevent severe COVID-19 outcomes among men.

## Key Points

**Question:**

Is male hypogonadism a risk factor for hospitalization for COVID-19?

**Findings:**

In this cohort study of 723 men, those with hypogonadism had significantly higher odds than men with eugonadism of being hospitalized, independent of other known risk factors for COVID-19–related hospitalization. Men receiving testosterone therapy had a similar risk of hospitalization as men with eugonadism.

**Meaning:**

This study suggests that men with hypogonadism are more likely to be hospitalized after COVID-19 infection compared with men with eugonadism and men receiving adequate testosterone therapy.

## Teaser

This cohort study compares COVID-19 hospitalization rates for men with hypogonadism who were not receiving testosterone therapy, men with eugonadism, and men receiving testosterone therapy.

## Introduction

The COVID-19 pandemic has exacted a heavy toll on public health. Epidemiologic data have identified certain characteristics that are associated with adverse outcomes, including advanced age, obesity, and systemic diseases, particularly diabetes, hypertension, chronic lung disease, and cardiovascular and cerebrovascular diseases.^1^ In addition, patients hospitalized for COVID-19 are more likely to be men than women.^2^ Therefore, it was presumed that testosterone is a risk factor for severe COVID-19 and that estrogen may be protective against COVID-19.^3^ However, not all men have similar testosterone concentrations.^4,5^ Men’s testosterone concentrations decrease continuously by 1% to 2% per year, starting after the third decade of life.^6,7,8^ In addition, obesity, metabolic syndrome, and chronic illnesses, such as type 2 diabetes, kidney insufficiency, and chronic lung disease, are associated with lower serum testosterone concentrations among men.^4,9,10^ Thus, aging and the presence of comorbid conditions, which are risk factors for hospitalization for COVID-19, are also associated with hypogonadism, which raises the question of whether hypogonadism is a risk factor for COVID-19–related hospitalization among men.

Recent studies have shown that testosterone concentrations during hospitalization are lower in men with severe COVID-19 than in men with a milder disease course.^11,12,13^ Because acute illness can lower testosterone concentrations,^14^ it was not clear whether testosterone concentrations in men with severe illness were subnormal even prior to contracting COVID-19. Men with chronically low testosterone concentrations have decreased muscle mass and less strength, both of which contribute to reduced lung capacity and ventilator dependence.^15,16,17^ Whether pre–COVID-19 testosterone concentrations in men are associated with the course of illness after COVID-19 remains unknown.

We reviewed the medical records of men who had a history of COVID-19 infection and had testosterone concentrations measured in the outpatient setting (when they did not have COVID-19). We hypothesized that men with subnormal testosterone concentrations prior to COVID-19 infection would be more likely to require hospitalization than men with normal testosterone concentrations. We also investigated whether testosterone therapy (TTh) modified the risk of hospitalization among men with hypogonadism.

## Methods

This retrospective cohort study was approved and a waiver of informed consent was granted by the institutional review boards of St Louis University and Washington University in St Louis, Missouri. The data set was deidentified after data collection, as per Health Insurance Portability and Accountability Act Authorization per section 164.512(i) of the Privacy Rule. This study followed the Strengthening the Reporting of Observational Studies in Epidemiology (STROBE) reporting guideline.

**Figure 1.**
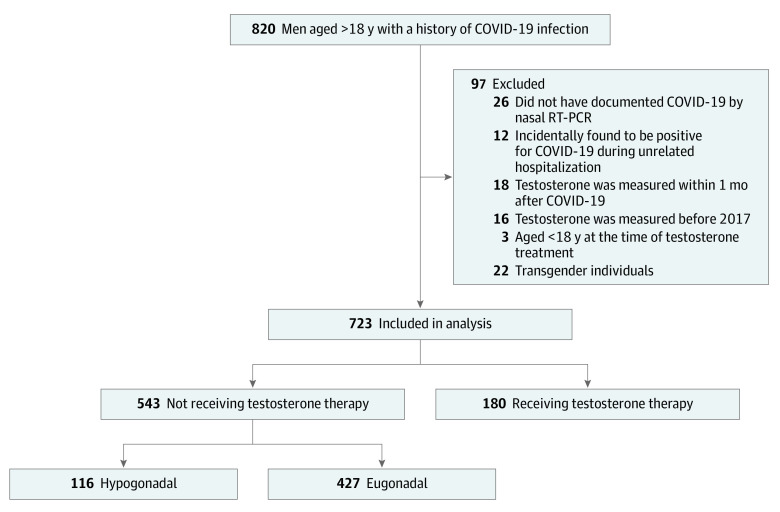
Study Sample and Group Allotment According to Study Inclusion and Exclusion Criteria RT-PCR indicates reverse transcription–polymerase chain reaction.

We searched the electronic health records of 2 major health systems in St Louis, SSM and BJC HealthCare, to identify patients who met the following criteria: men, older than 18 years of age, history of COVID-19 infection (*International Statistical Classification of Diseases and Related Health Problems, Tenth Revision* code U07.1), and had at least 1 measurement of testosterone concentration. The study period was between January 1, 2017, and December 31, 2021, and 820 patients met the inclusion criteria (Figure 1). The electronic health records of these patients were individually reviewed. We excluded patients whose COVID-19 had not been documented by a positive nasal reverse transcription–polymerase chain reaction test or who were incidentally found to be positive for COVID-19 during screening for a planned surgical procedure or unrelated hospitalization. If multiple testosterone measurements were available, we chose the one that was collected in an outpatient setting prior to COVID-19 infection, in the morning (if available), and temporally closer to the COVID-19 infection. We excluded patients whose testosterone concentrations were measured only during an acute illness (such as hospitalization). Other exclusion criteria were (1) testosterone concentrations measured less than 1 month after COVID-19, (2) age younger than 18 years at the time of testosterone measurement, and (3) transgender individuals. Testosterone concentrations prior to January 2017 (>5 years ago) were excluded because they might not have reflected the baseline gonadal state of the patient during the COVID-19 infection. After review of electronic health records, 97 men were found to be ineligible based on the study criteria. Thus, 723 men were included in the final analysis.

We collected information on demographic characteristics (age, body mass index [BMI; calculated as weight in kilograms divided by height in meters squared], and race and ethnicity), comorbid conditions, hospitalization for COVID-19, and receipt of TTh. For inclusion in the TTh group, a patient should have been receiving TTh for at least 6 months preceding the COVID-19 infection. Patients were presumed to be immunosuppressed if they had an organ transplant or were receiving supraphysiological doses of corticosteroids or taking immunosuppressive medications for other conditions, such as multiple sclerosis, inflammatory bowel disease, rheumatologic diseases, or malignant neoplasm. For hospitalized patients, we collected information on the duration of hospitalization, intensive care unit (ICU) admission, use of a ventilator, and number of days on a ventilator. We also collected data on mortality and assessed whether the death was associated with COVID-19. All of the medical records with ambiguity regarding the assessment of data were audited by the first author (S.D.).

Outpatient testosterone measurements were ordered owing to symptoms suggestive of hypogonadism. Fatigue was the most common symptom (present in 43% of patients [311 of 723]), followed by erectile dysfunction (30% [217 of 723]). Other reasons included decreased libido (8% [58 of 723]), gynecomastia (5% [39 of 723]), low mood (4% [29 of 723]), opiate use (3% [24 of 723]), pituitary adenoma (3% [23 of 723]), and obesity (3% [22 of 723]). The measurements were performed using immunoassays (for 61% of patients [441 of 723]) or by the criterion standard method, liquid chromatography tandem mass spectrometry (for 39% of patients [282 of 723]) by commercial laboratories. The lower limit of normal for testosterone concentrations in the laboratories varied between 175 and 300 ng/dL (to convert to nanomoles per liter, multiply by 0.0347). The upper limit ranged from 716 to 1100 ng/dL. We defined hypogonadism as a total testosterone concentration below the lower limit of normal in the reference range provided by the laboratory.

### Statistical Analysis

The primary exposure of the study was the gonadal status (hypogonadism, eugonadism, and TTh), and the primary outcome was hospitalization for COVID-19. Statistical adjustments were made for group differences in age, BMI, race and ethnicity (coded as Black, White, or other [Asian, Pacific Islander, or American Indian]), immunosuppression (yes or no), and comorbid conditions (Charlson Comorbidity Index [CCI]). Racial disparities have been noted in outcomes of COVID-19. Hence, we included race and ethnicity as a covariate in our analysis. The CCI is a validated method to assess significant medical comorbidity.^18^ Continuous variables are presented as either mean (SD) values or median (IQR) values, depending on the distribution of values. Testosterone concentrations were not normally distributed and were log-transformed to conduct parametric tests. All tests were performed using SPSS software, version 27 (SPSS Inc). Group comparisons were performed by *t* tests, analysis of variance (with Hochberg post hoc analyses), Mann-Whitney rank sum tests, χ^2^ tests, and Fisher exact tests as appropriate. Multivariable logistic regression analyses are presented as odds ratios (ORs [ie, exponential of the β coefficient with 95% CIs and *P* values]). Reported *P* values are 2-sided and considered statistically significant at *P* < .05. Our study had 90% power to detect a 15% difference in hospitalization rates between the group with eugonadism and the group with hypogonadism.

## Results

The mean (SD) age of the 723 participants was 55 (14) years, the mean (SD) BMI was 33.5 (7.3), 116 men had hypogonadism, 427 had eugonadism, and 180 were receiving TTh. Testosterone concentrations were measured prior to COVID-19 in 73% of patients (525 of 723). The median time between the testosterone measurement and COVID-19 infection was 7 months (IQR, 3-16 months). For 27% of patients (198 of 723), testosterone concentrations were available only after recovery from COVID-19; the median time between COVID-19 and testosterone measurement among those patients was 6 months (IQR, 3-9 months).

The incidences of COVID-19 infection and hospitalization are shown in eFigures 1 and 2 in the Supplement. Overall, 134 men were hospitalized owing to COVID-19. Men who required hospitalization were older (mean [SD] age, 62 [15] vs 53 [14] years), had more comorbid conditions (median CCI, 2 [IQR, 0-3] vs 0 [IQR, 0-1], and were more likely to be immunosuppressed (26 of 134 [19%] vs 24 of 589 [4%]) than men who were not hospitalized (eTable in the Supplement).

As shown in Table 1, men with hypogonadism were more likely to require hospitalization than those with eugonadism (52 of 116 [45%] vs 53 of 427 [12%]). Men receiving TTh had a risk of hospitalization similar to those with eugonadism (29 of 180 [16%] vs 53 of 427 [12%]). Men with hypogonadism had a higher risk of ICU admission than those with eugonadism (10 of 116 [9%] vs 13 of 427 [3%]) but a similar risk of ventilator requirement (5 of 116 [4%] vs 7 of 427 [2%]) and mortality (5 of 116 [4%] vs 9 of 427 [2%]). However, very few men experienced these outcomes. There were no differences among the men with hypogonadism, the men with eugonadism, and the men receiving TTh in the median number of days spent in the hospital (6 days [IQR, 3-12 days] vs 6 days [IQR, 3-10 days] vs 8 days [IQR, 4-13 days]), ICU (6 days [IQR, 4-24 days] vs 4 days [IQR, 2-17 days] vs 14 days [IQR, 6-17 days]), or on ventilators (14 days [IQR, 3-44 days] vs 8 days [IQR, 4-44 days] vs 12 days [IQR, 4-16 days]).

**Table 1.** Demographic and Hospitalization Characteristics

Characteristic	Men, No. (%)			*P* value
	Hypogonadal (n = 116)	Eugonadal (n = 427)	Testosterone therapy (n = 180)	
Age, mean (SD), y	62 (15)^a^^,^^b^	53 (14)	55 (12)	<.001
BMI, mean (SD)	35 (9)^a^	33 (7)^b^	35 (7)^a^	<.001
Obesity	73 (63)	259 (61)^b^	129 (72)	.04
Testosterone, median (IQR), ng/dL	131 (18-187)^a^^,^^b^	381 (300-504)	396 (248-624)	<.001
Charlson comorbidity index, median (IQR)	2 (0-3)	0 (0-1)	0 (0-2)	<.001
Diabetes	45 (39)	89 (21)	57 (32)	<.001
Immunosuppressed	19 (16)	24 (6)	7 (4)	<.001
History of cardiovascular events	24 (21)^a^	48 (11)	24 (13)	.03
Race and ethnicity				
Black	26 (22)^a^^,^^b^	62 (15)^b^	12 (7)	<.001
White	87 (75)^b^	353 (83)^b^	167 (93)	<.001
Other^c^	3 (3)	12 (3)	1 (1)	.22
Hospitalized	52 (45)^a^^,^^b^	53 (12)	29 (16)	<.001
ICU	10 (9)^a^	13 (3)	9 (5)	.03
Ventilator	5 (4)	7 (2)	6 (3)	.18
Mortality from COVID-19	5 (4)	9 (2)	3 (2)	.30
Length of stay in hospital, median (IQR), d	6 (3-12)	6 (3-10)	8 (4-13)	.33
Length of stay in ICU, median (IQR), d	6 (4-24)	4 (2-17)	14 (6-17)	.39
Time on ventilator, median (IQR), d	14 (3-44)	8 (4-44)	12 (4-16)	.90

Abbreviations: BMI, body mass index (calculated as weight in kilograms divided by height in meters squared); ICU, intensive care unit.

SI conversion factor: To convert testosterone to nanomoles per liter, multiply by 0.0347.

^a^ *P* < .05 compared with the group with eugonadism.

^b^ *P* < .05 compared with the group receiving TTh.

^c^ Asian, Pacific Islander, or American Indian.

We calculated the odds ratios of hospitalization among the 3 groups after adjustment for age, BMI, race and ethnicity, CCI, and immunosuppression rates (Table 2).^19^ Men with hypogonadism were 2.4 times more likely than those with eugonadism to require hospitalization for COVID-19 in the fully adjusted model (OR, 2.4; 95% CI, 1.4-4.4; *P* < .003). Men receiving TTh had a similar risk of hospitalization as men with eugonadism (OR, 1.3; 95% CI, 0.7-2.3; *P* = .35). Other significant factors associated with hospitalization in the adjusted model were age (OR, 1.03; 95% CI, 1.01-1.05; *P* = .02), CCI (OR, 1.3; 95% CI, 1.1-1.5; *P* < .001), and immunosuppression (OR, 3.5; 95% CI, 1.5-7.8; *P* = .003). Men with hypogonadism were not more likely than those with eugonadism to require ICU admission after multivariable adjustment (OR, 1.2; 95% CI, 0.4-3.5; *P* = .69).

**Table 2.** Odds Ratios of Hospitalization Risk Among Men With Hypogonadism and Men Receiving TTh Compared With Men With Eugonadism

Model	Eugonadal	Hypogonadal		TTh	
		Odds ratio (95% CI)	*P* value	Odds ratio (95% CI)	*P* value
Unadjusted	1 [Reference]	5.7 (3.6-9.1)	<.001	1.4 (0.8-2.2)	.23
Adjusted for age, BMI, immunosuppression status, race and ethnicity, and CCI^a^	1 [Reference]	2.4 (1.4-4.4)	<.003	1.3 (0.7-2.3)	.35

Abbreviations: BMI, body mass index (calculated as weight in kilograms divided by height in meters squared); CCI, Charlson Comorbidity Index; TTh, testosterone therapy.

^a^ The estimated relative risks in the fully adjusted model for the group with hypogonadism and the group receiving TTh were 2.1 (95% CI, 1.3-3.1) and 1.3 (95% CI, 0.7-2.0), respectively, compared with the group with eugonadism. The relative risks were derived from odds ratios.^19^

### Testosterone Concentrations and Risk of Hospitalization Among Men With Hypogonadism and Men With Eugonadism

**Figure 2.**
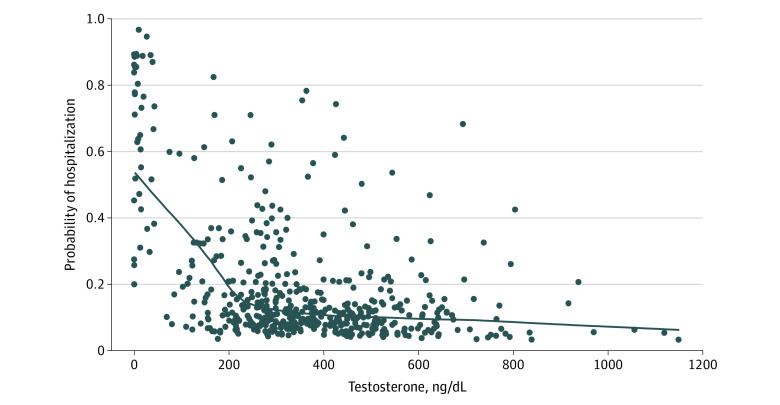
Probability of Hospitalization Based on Testosterone Concentrations in Men With Hypogonadism and Men With Eugonadism, After Multivariable Adjustment for Age, Body Mass Index, Charlson Comorbidity Index, Race and Ethnicity, and Immunosuppression Status SI conversion factor: To convert testosterone to nanomoles per liter, multiply by 0.0347.

We compared the risk of hospitalization across the continuum of testosterone concentrations. Hospitalization rates increased if the patient’s testosterone concentration was lower than 200 ng/dL (Figure 2). Thirty-two men were receiving androgen deprivation therapy for prostate cancer; their median testosterone concentration was 3.5 ng/dL (IQR, 1.5-14.8 ng/dL). Eighteen of those men (56%) were hospitalized owing to COVID-19, and 3 men (9%) required ICU care.

Testosterone concentrations were measured before COVID-19 among 67% of men with eugonadism (284 of 427) and 75% of men with hypogonadism (87 of 116). We considered the possibility that COVID-19 illness may have induced a long-term change in testosterone concentrations. Hence, we compared the ORs for hospitalization separately among patients whose testosterone concentrations had been measured before or after COVID. The ORs were similar to the overall results; the ORs for hospitalization were 2.5 (95% CI, 1.2-5.0; *P* = .01) among patients whose testosterone concentrations were measured before COVID-19 and 3.6 (95% CI, 1.0-13.2; *P* = .051) among patients whose testosterone concentrations had been measured after COVID-19. We also compared the testosterone concentrations of men whose concentrations were measured before COVID-19 (n = 371) with those after COVID-19 (n = 172). The median testosterone concentrations were similar in both groups (before COVID-19, 325 ng/dL [IQR, 232-458 ng/dL]; after COVID-19, 366 ng/dL [IQR, 261-484 ng/dL]; *P* = .22 after adjustment for age, BMI, and CCI). The prevalence of hypogonadism among men whose testosterone concentrations were measured before COVID-19 was similar to that among men whose testosterone concentrations were measured after COVID-19 (before, 86 of 371 [23%]; after, 29 of 172 [17%]; *P* = .08).

Testosterone concentrations were measured in the morning for 77% of men with hypogonadism (89 of 116) and 79% of men with eugonadism (337 of 427). Among the remaining men, testosterone concentrations had been measured only after 12 PM. Because testosterone concentrations are lower in the evening in men owing to diurnal variation, we reanalyzed the risk of hospitalization after eliminating the 23% of patients with hypogonadism (27 of 116) whose testosterone concentrations were measured in the evening. Similar to the whole population, men with hypogonadism had higher odds of hospitalization than men with eugonadism (multivariable adjusted OR, 2.0; 95% CI, 1.0-3.8; *P* = .04).

### Hospitalization Among Men Receiving TTh

The most frequent mode of TTh was intramuscular testosterone cypionate or enanthate injections (69% [124 of 180]), followed by topical gels (29% [52 of 180]) and patches (2% [4 of 180]). No patients were receiving long-acting intramuscular testosterone undecanoate, oral testosterone undecanoate, insertable pellets, or other modalities of testosterone replacement. The median testosterone concentration in men receiving TTh was similar to that in men with eugonadism (396 ng/dL [IQR, 248-624 ng/dL] vs 381 ng/dL [IQR, 300-504 ng/dL]) (Table 1). However, 44 of 180 men (24%) had subnormal testosterone concentrations while receiving TTh. These men had higher odds of hospitalization (OR, 3.5; 95% CI, 1.5-8.6; *P* = .003, adjusted for age, BMI, CCI, race and ethnicity, and immunosuppression) compared with men who had normal testosterone concentrations while receiving TTh.

## Discussion

We found that men with hypogonadism were 2.4 times more likely than men with eugonadism to be hospitalized if they contracted COVID-19. This increased risk was independent of other factors that increase the risk of hospitalization for COVID-19, such as advanced age, comorbid conditions, and immunosuppression. This finding contrasts with a widely held notion that men are more likely than women to be admitted owing to COVID-19 because they have higher concentrations of circulating testosterone. On the contrary, our data suggest that male hypogonadism is a risk factor for hospitalization for COVID-19.

A previous study measured testosterone concentrations in hospitalized men admitted with COVID-19 and found that 89% had subnormal testosterone concentrations on admission to the hospital.^11^ Men with a more severe course of COVID-19 had lower testosterone concentrations than men with a milder course. Similar findings have been reported in studies all around the world.^13,20^ It is now well established that a low testosterone concentration is a marker of severe illness after COVID-19, presumably owing to a suppressive effect of acute illness on the hypothalamic-pituitary-gonadal axis.^14^ However, it was not clear whether testosterone concentrations measured when the men did not have COVID-19 had any bearing on the course of their COVID-19 illness. Our analysis shows that long-term low testosterone concentrations may be associated with hospitalization for COVID-19 among men. It is possible that testosterone concentrations serve as a marker for general health. Some of the risk factors for COVID-19–related hospitalization, such as advanced age, obesity, and comorbid conditions, are also associated with male hypogonadism.^4,21^ However, our results were robust for adjustment for these covariates. Hence, it is likely that testosterone is an independent factor associated with the course of COVID-19 illness among men. Our findings have important implications because hypogonadism is common among men, and physicians encounter this condition almost daily in their outpatient practice. The prevalence of hypogonadism among men varies from 10% to 40% depending on age, BMI, and presence of comorbid conditions.^5,22,23^ However, hypogonadism remains undiagnosed and untreated among many men.

We also found that men receiving TTh were less likely to be hospitalized for COVID-19 than men with hypogonadism who were not receiving TTh. The risk of hospitalization among men receiving TTh was similar to that among men with eugonadism. This risk reduction was limited to men who achieved normal testosterone concentrations while receiving TTh. Men receiving inadequate TTh had a higher risk of hospitalization than those receiving adequate TTh. The reasons for inadequate TTh were not always recorded in the medical records, but common reasons for inadequate TTh were prescription coverage, cost of therapy, lack of refills, patient adherence to therapy, and missed clinic appointments. The rate of inadequate TTh in our study is similar to the rates observed in other observational studies.^24,25,26^ A large database study of 69 632 patients receiving TTh at Veterans Health Administration clinics found that 37% had subnormal testosterone concentrations.^26^ These studies found that the health benefits associated with TTh were restricted to those receiving adequate TTh. The reduction in the hospitalization rate with TTh suggests that testosterone is not simply a marker of chronic health. Rather, these findings suggest a mediator role for testosterone. Men with hypogonadism have decreased muscle mass and strength, which is associated with functional decline and repeated hospitalizations.^27^ TTh is known to increase muscle mass and strength.^28^ Epidemiological studies have found that TTh was associated with decreased readmission after hospitalization among elderly men.^29^ TTh was also associated with reduced hospitalizations for respiratory conditions among men with chronic obstructive pulmonary disease.^30^ Men with hypogonadism are in a generalized inflammatory state compared with men with eugonadism, and TTh reduces serum inflammatory mediators and their action at the cellular level.^31,32,33^ One small study conducted medical record reviews of 32 men receiving TTh and compared them with 63 matched patients not receiving TTh.^34^ The study found similar hospitalization and ICU admission rates after COVID-19 between the 2 groups. These results are consistent with our findings.

SARS-CoV-2 binds to angiotensin-converting enzyme 2 receptors and undergoes S protein priming by the type II transmembrane serine protease (TMPRSS2) to enter cells.^35,36^ Because TMPRSS2 is regulated by the androgen receptor,^37^ it has been hypothesized that androgen depletion or blockade can be a therapeutic strategy to minimize COVID-19 illness. Contrary to this hypothesis, a randomized clinical trial found that androgen blockade with enzalutamide for hospitalized patients with COVID-19 resulted in longer hospital stays and no benefit compared with standard of care.^38^ Epidemiological studies that evaluated the severity of COVID-19 infections in men receiving androgen deprivation therapies have shown inconsistent results and do not support androgen deprivation therapy as a protection against COVID-19.^39^ We found a high rate of hospitalization (56%) among men receiving androgen deprivation therapies in our study. The association between hospitalizations and testosterone concentrations appeared to be inversely linear below 200 ng/dL, with no indication of benefit at very low testosterone concentrations.

### Limitations

Our study has many limitations, including an uneven distribution of confounding variables between groups, which required statistical adjustments. A low event rate for death, ICU care, or ventilator therapy precluded assessment of hypogonadism as a risk factor for severe COVID-19. Also, because most hospitalizations were during the first year of the pandemic, when COVID-19 vaccines were not available (eFigure 2 in the Supplement), it is unclear whether the findings apply to patients who are fully vaccinated. The decreasing prevalence of COVID-19 and the widespread availability of vaccines imply that a prospective study to evaluate the association of hypogonadism and TTh with the natural course of COVID-19 illness may not be feasible, unless a new vaccine-resistant variant of COVID-19 were to emerge. Our retrospective study, therefore, provides insights to explore the role of testosterone in other acute respiratory illnesses. Testosterone concentrations were not available prior to COVID-19 for one-third of the men with hypogonadism in our study. However, we did not find a difference in hypogonadism prevalence or COVID-19 hospitalization risk among cohorts with testosterone measurements available before or after COVID-19. In addition, COVID-19 infection did not result in a permanent lowering of testosterone, thyroid, or cortisol concentrations in prior studies.^40,41^ Unbound testosterone concentrations were either not available or were measured by inaccurate methods for most patients. Total testosterone concentrations were not always measured by mass spectrometry.^21,42^ Ideally, the diagnosis of hypogonadism requires 2 subnormal testosterone measurements because of the day-to-day variation in testosterone concentrations.^42^ However, the probability of a change in either direction with repeated measurements is statistically equal. Therefore, while repeated measurements are important for the diagnosis of hypogonadism in a given patient, most epidemiologic or cross-sectional studies rely on a single measure of testosterone concentration.^23^ Therefore, we used a single measure of testosterone conentration to document the adequacy of TTh, as previously described.^26^ Although it is possible that some men with eugonadism were included in the group with hypogonadism, such misclassification would have led to a type II error (false-negative result). Hence, the availability of 2 measures of testosterone concentration would have likely amplified the differences in hospitalization among the men with hypogonadism and those with eugonadism.

## Conclusions

The findings of this cohort study suggest that men with hypogonadism are more likely than men with eugonadism to be hospitalized after COVID-19 infection. They also suggest that men with hypogonadism who receive adequate TTh to achieve normal testosterone concentrations have a reduction in the risk of COVID-19–related hospitalization. Prospective clinical trials are needed to explore the efficacy of TTh in preventing hospitalizations after COVID-19 and similar respiratory illnesses among men with hypogonadism.

## Notes

Supplement. **eTable.** Comparison of Patients That Were Hospitalized or Not Hospitalized Following COVID-19 Infection **eFigure 1.** Number of Patients With COVID-19 (Blue Line, Black Circles) and Patients Hospitalized (Red Broken Line, White Circles) Over the Study Duration **eFigure 2.** Percentage of Patients Hospitalized With COVID-19
