# Supplementary material for: Association of Male Hypogonadism With Risk of Hospitalization for COVID-19
Source: JAMA Netw Open. 2022 Sep 2;5(9):e2229747. doi: 10.1001/jamanetworkopen.2022.29747 (PMC9440397; doi:10.1001/jamanetworkopen.2022.29747)
Supplement: Supplement. — eTable. Comparison of Patients That Were Hospitalized or Not Hospitalized Following COVID-19 Infection eFigure 1. Number of Patients With COVID-19 (Blue Line, Black Circles) and Patients Hospitalized (Red Broken Line, White Circles) Over the Study Duration eFigure 2. Percentage of Patients Hospitalized With COVID-19 [file jamanetwopen-e2229747-s001.pdf]

Supplementary Online Content

Dhindsa S, Champion C, Deol E, et al. Association of male hypogonadism with risk of hospitalization for COVID-19. *JAMA Netw Open.* 2022;5(9):e2229747. doi:10.1001/jamanetworkopen.2022.29747

eTable. Comparison of Patients That Were Hospitalized or Not Hospitalized Following COVID-19 Infection
eFigure 1. Number of Patients With COVID-19 (Blue Line, Black Circles) and Patients Hospitalized (Red Broken Line, White Circles) Over the Study Duration
eFigure 2. Percentage of Patients Hospitalized With COVID-19

This supplementary material has been provided by the authors to give readers additional information about their work.

© 2022 Dhindsa S et al. *JAMA Network Open.*

eTable. Comparison of Patients That Were Hospitalized or Not Hospitalized Following COVID-19 Infection. Charlson comorbidity index and length of stay are median [25th, 75th percentile].

		Hospitalized (n=134)	Not hospitalized (n= 589)	P
Age (years)		62±15	53±14	< .001
BMI (kg/m^2)		33±8	34±7	.15
Obesity (n, %)		79, 59%	382, 65%	.20
Charlson comorbidity index		2 [0, 3]	0 [0, 1]	< .001
Diabetes (n, %)		64, 47%	127, 22%	< .001
History of cardiovascular events (n, %)		30, 22%	66, 11%	< .001
Race	Black	30, 23%	70, 12%	.001
	White	100, 74%	507, 86%	.001
	Other[a]	4, 3%	12, 2%	.51
Immunosuppressed (n, %)		26, 19%	24, 4%	< .001
ICU (n, %)		32, 24%	N/A	
Ventilator (n, %)		18, 13%	N/A	
Mortality due to COVID (n, %)		16, 12%	1, 0.2%	< .001
Length of stay in hospital (days)		6 [3, 11]	N/A	

N/A: not applicable
[a]Asian, Pacific Islander, Native American

© 2022 Dhindsa S et al. *JAMA Network Open.*

eFigure 1. Number of Patients With COVID-19 (Blue Line, Black Circles) and Patients Hospitalized (Red Broken Line, White Circles) Over the Study Duration. The highest number of documented COVID-19 infections was in the last 3 months of 2020. This coincides with data on average cases published by the Centers for Disease Control (https://covid.cdc.gov/covid-data-tracker/#trends_dailycases).

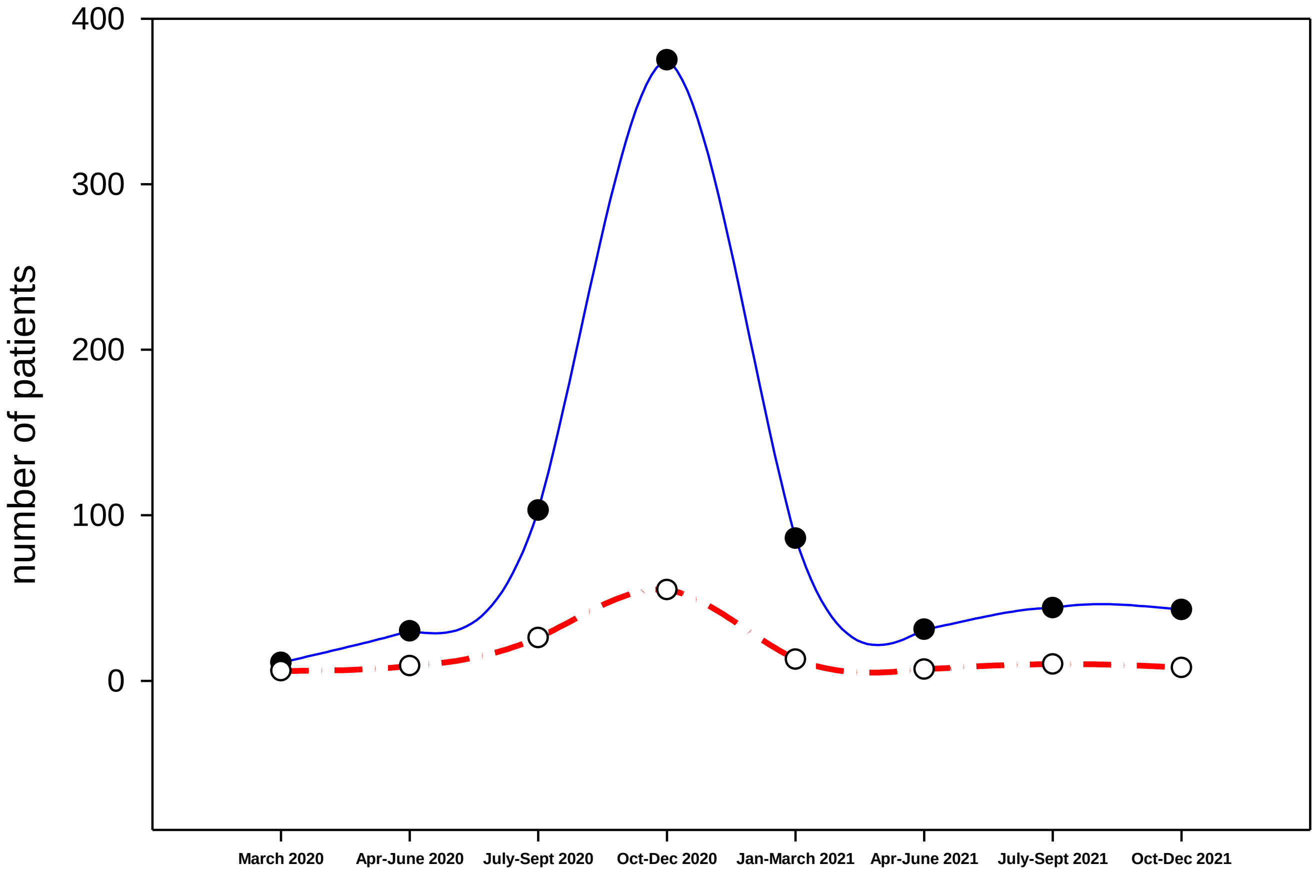

© 2022 Dhindsa S et al. *JAMA Network Open.*

eFigure 2. Percentage of Patients Hospitalized With COVID-19. The hospitalization rates were very high in the first 4 months of the pandemic but stabilized thereafter to ~15% through 2020 and 2021.

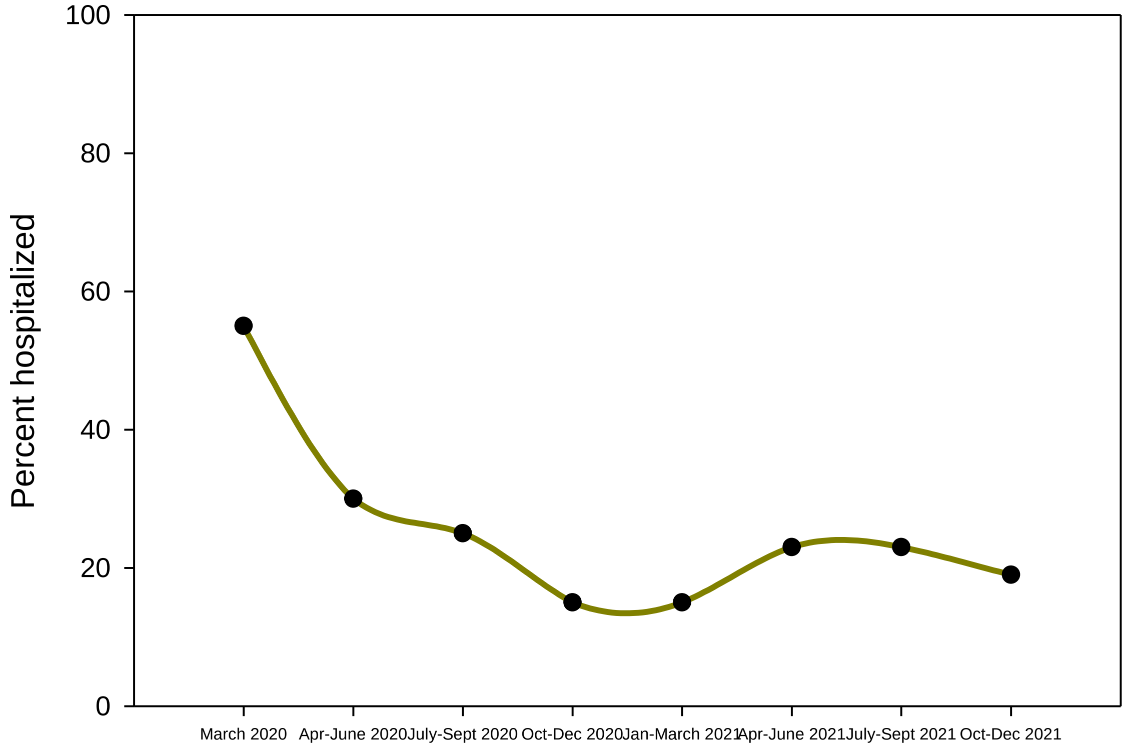

© 2022 Dhindsa S et al. *JAMA Network Open.*
